# Hidden Diversity Behind the Zombie-Ant Fungus *Ophiocordyceps unilateralis*: Four New Species Described from Carpenter Ants in Minas Gerais, Brazil

**DOI:** 10.1371/journal.pone.0017024

**Published:** 2011-03-02

**Authors:** Harry C. Evans, Simon L. Elliot, David P. Hughes

**Affiliations:** 1 Department of Animal Biology, Federal University of Viçosa (UFV), Minas Gerais, Brazil; 2 CAB International, E-UK, Egham, Surrey, United Kingdom; 3 Department of Entomology and Department of Biology, Penn State University, University Park, Pennsylvania, United States of America; 4 School of Biosciences, University of Exeter, Exeter, United Kingdom; Field Museum of Natural History, United States of America

## Abstract

**Background:**

*Ophiocordyceps unilateralis* (*Clavicipitaceae: Hypocreales*) is a fungal pathogen specific to ants of the tribe *Camponotini* (Formicinae: Formicidae) with a pantropical distribution. This so-called zombie or brain-manipulating fungus alters the behaviour of the ant host, causing it to die in an exposed position, typically clinging onto and biting into the adaxial surface of shrub leaves. We (HCE and DPH) are currently undertaking a worldwide survey to assess the taxonomy and ecology of this highly variable species.

**Methods:**

We formally describe and name four new species belonging to the *O. unilateralis* species complex collected from remnant Atlantic rainforest in the south-eastern region (Zona da Mata) of the State of Minas Gerais, Brazil. Fully illustrated descriptions of both the asexual (anamorph) and sexual (teleomorph) stages are provided for each species. The new names are registered in Index Fungorum (registration.indexfungorum.org) and have received IF numbers. This paper is also a test case for the electronic publication of new names in mycology.

**Conclusions:**

We are only just beginning to understand the taxonomy and ecology of the *Ophiocordyceps unilateralis* species complex associated with carpenter ants; macroscopically characterised by a single stalk arising from the dorsal neck region of the ant host on which the anamorph occupies the terminal region and the teleomorph occurs as lateral cushions or plates. Each of the four ant species collected - *Camponotus rufipes*, *C. balzani*, *C. melanoticus* and *C. novogranadensis* - is attacked by a distinct species of *Ophiocordyceps* readily separated using traditional micromorphology. The new taxa are named according to their ant host.

## Introduction

The genus *Cordyceps* Fr. (1818) was established to accommodate ascomycete fungal pathogens of arthropods bearing the sexual spore (ascospore)-producing structures (ascomata) in or on conspicuous stalks (stromata) arising from the host cadaver. The polyphyletic nature of the genus had been recognised for some time [1], [2] - especially when parasites of truffle-like fungi (*Elaphomyces*) were included subsequently in the genus - but only recently has this been conclusively confirmed following a multi-gene study [3]. Species formerly assigned to the genus now occupy four genera in three families of the order *Hypocreales*.

*Ophiocordyceps unilateralis* (Tul.) Petch - a ubiquitous pathogen of ants with a pantropical distribution and more commonly known under the name of *Cordyceps unilateralis* (Tul.) Sacc. - was reinstated in the genus *Ophiocordyceps* [3], originally erected to accommodate species with non-fragmenting ascospores [1]. This was designated the type genus of the new family *Ophiocordycipitacae*, and currently comprises around 140 species [3]. It was argued that: “Because *O. unilateralis* is a well-known species that was included in the original publication of *Ophiocordyceps* and because additional *Ophiocordyceps* species are members of this clade, we apply the name *Ophiocordyceps* based on the placement of *O. unilateralis*” [3]. This species, therefore, is central to our understanding of the taxonomy and functional morphology of this ecologically important group of fungi.

**Figure 1:**
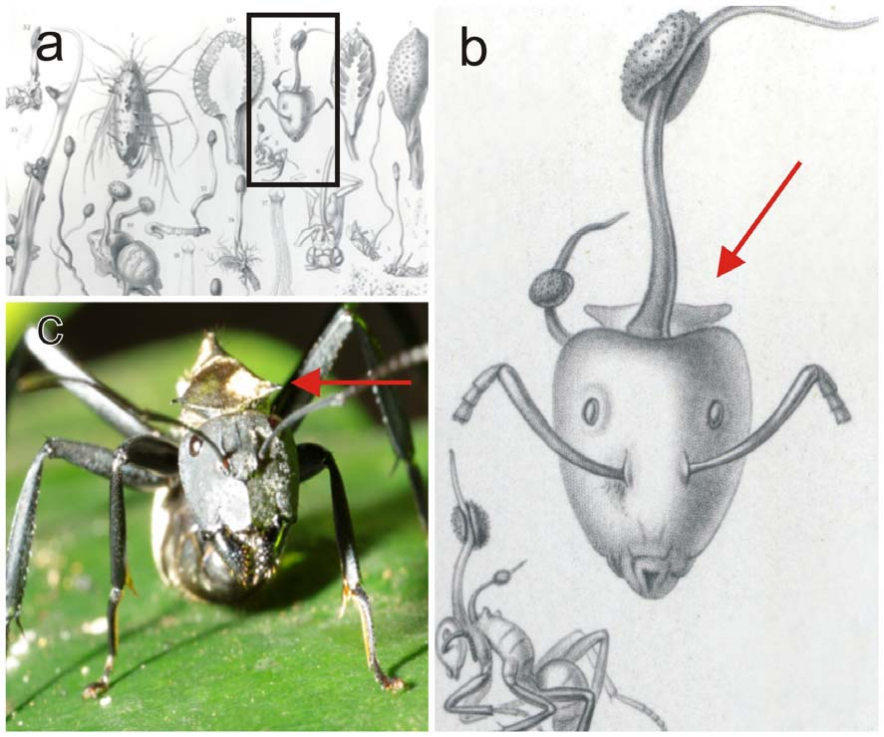
*Ophiocordyceps unilateralis*. **a**) Original plate from the 1865 *Selecta Fungorum Carpologia* of the Tulasne brothers [4], illustrating the holotype of *Ophiocordyceps* (*Torrubia*) *unilateralis* and said to be on the leaf-cutting ant, *Atta cephalotes*; **b**) Detail from plate showing the distinctive pronotal plate of *Camponotus sericeiventris*, as well as a side view of the host which is clearly a carpenter ant and not a leaf-cutter; compare with **c**) Live worker of *C. sericeiventris* showing the spines on the pronotal plate (arrow).

Perversely, the taxonomy of *O. unilateralis* remains unclear, since apparently, the type specimen is immature and the salient morphological features – ascomata, asci and ascospores – were never included in the original description of *Torrubia unilateralis* Tul. in 1865 [4]: later confirmed by Y. Kobayasi who examined the holotype held in the Museum of Entomology in Paris [2]. L. Tulasne listed the host as the leaf-cutting ant *Atta cephalotes* from Brazil, and the specimen is beautifully illustrated by his brother C. Tulasne [4; see Fig. 1a–b]: who is noted for the accuracy of his drawings. Clearly, the ant host depicted is not a leaf-cutter and strongly resembles a carpenter ant, specifically *Camponotus sericeiventris* Mayr based on the distinctive pronotal plate (Stefan Cover, Museum of Comparative Zoology, Harvard Museum, pers. comm., May 2010; see Fig. 1c). In addition, after more than 150 years of collecting, *O. unilateralis* has only ever been recorded on ant hosts of the tribe *Camponotini*, whilst leaf-cutting ants appear to be remarkably free from infections by entomopathogenic fungi with few confirmed associations [5], [6]. Unfortunately, the type specimen is not available for examination since, thus far, it cannot be located at the Paris Museum (Bart Buyck, pers. comm., August 2010). Whether or not the type specimen was actually returned to France from Japan is a moot point since this coincided with the chaotic events of the early stages of World War II. Priority is being given to finding a neotype and on-going searches in Brazil have found two specimens of infected *Camponotus sericeiventris*, both of which were immature.

We set out to explore the taxonomy, as well as the ecology, of this keystone species because of uncertainties about its status, especially the levels of variation in morphology that had been noted over the years from collections worldwide [1], [2], [4], [7], [8]. Our hypothesis rested on the assumption that this variation was due to geographic or host isolation and that this would probably be best interpreted at the varietal level, as reported previously amongst the ‘*Cordyceps*’ species associated with ants [2], [8], [9], [10]. Our ‘World Tour’ began in Brazil, investigating the pathogens of carpenter ants in the Atlantic rainforest system of the south-eastern region of Minas Gerais State, locally known as the Zona da Mata. Here we report on these initial findings, using ‘living’ rather than herbarium specimens, that conclusively reject the theory that variation within the so-called *O. unilateralis* complex is the result of geographic isolation – and, moreover, that variation would involve only minor morphological characters that could be interpreted at the varietal level - and describe four new taxa from different species of *Camponotus*, all collected within a small area of fragmented, remnant forest. Differences in the functional morphology of all the spore stages within each species reflect the ecology of the ant host, and these are so pronounced that molecular characterisation is not required to separate them. However, DNA has been recovered from the new species and will be sequenced together with planned collections from across the tropics to obtain an overall picture of diversity within *O. unilateralis s.l.*

## Results and Discussion

### Taxonomic treatment

***Ophiocordyceps camponoti-rufipedis*** H.C. Evans & D.P. Hughes sp.nov.

**IF 550001**

http://www.indexfungorum.org/Names/NamesRecord.asp?RecordID=550001

Type: Brazil. Minas Gerais: Viçosa, Mata do Paraíso, 700 m, 16 Mar 2010, *H.C. Evans*, *D.P. Hughes & S.L. Elliot*, MAP-16, on *Camponotus rufipes* (Fabricius) (holotype IMI 399088; isotype VIC 31424).

Stromata semper singula, pronoto camponoti rufipedis oriunda; pars fertilis lateralis, 1–3, disciformis ad hemisphaerica, castaneus, amplitudine variabilis, av. 1.0×0.5 mm usque 2 mm longis. Ascomata in massa densa hyphali immersa vel parum erumpentia, ostiolis expositis, lageniformia, 175–260×100–130 µm. Asci octospori, hyalini, cylindrici vel clavati, (110–) 120–160×(6–) 8–10 µm; pileo inspissato terminati, 4–5.5×3–4.5 µm. Ascosporae hyalinae, vermiformes, (75–) 80–95 (–115)×2–3 µm, 4–7-septatae, non secedentes in cellulas.

**Figure 2:**
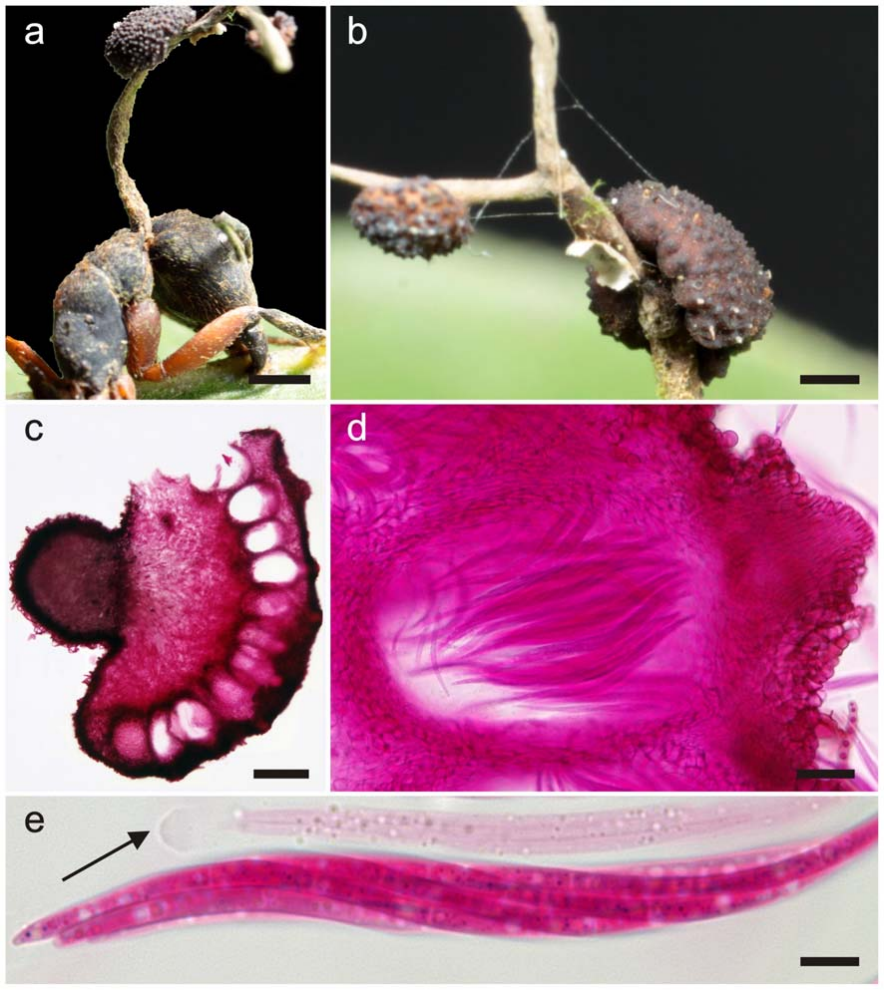
*Ophiocordyceps camponoti-rufipedis*. **a**) Single stroma, characteristic of *Ophiocordyceps unilateralis sensu lato*, with two lateral ascomatal cushions or plates arising from the dorsal pronotum of *Camponotus rufipes* (the red-legged ant), firmly attached to a leaf vein (bar  = 0.8 mm); **b**) Detail of fertile region showing the immersed to partially erumpent ascomata within the cushions, with the short necks or ostioles visible (bar  = 0.4 mm); **c**) Section through an ascomatal cushion showing the mainly immersed arrangement of ascomata (bar  = 150 µm), and detail of asci within chamber (**d**, bar  = 25 µm); **e**) Asci, clavate in shape and with prominent refractive cap (arrow, bar  = 7.5 µm).

**Figure 3:**
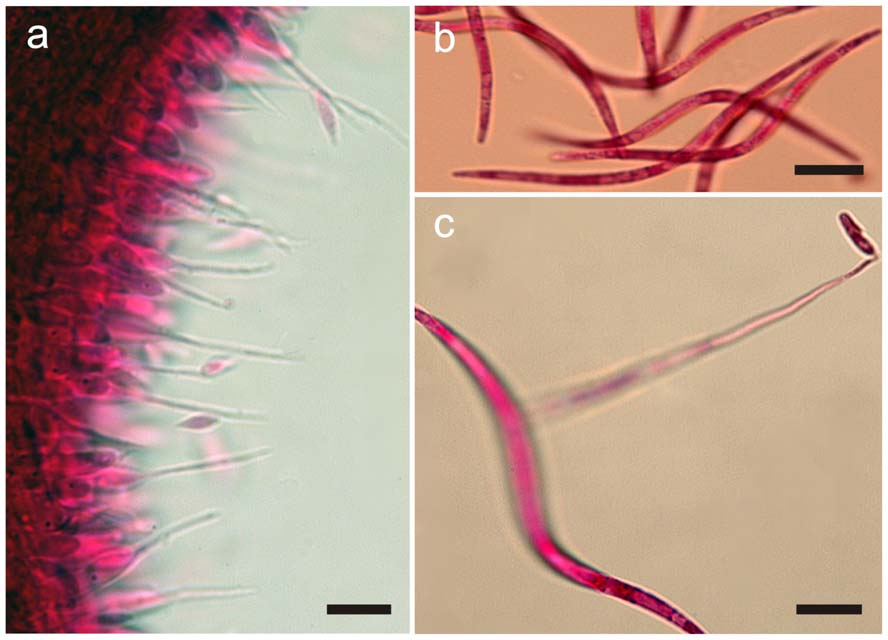
*Ophiocordyceps camponoti-rufipedis*. **a**) Section of upper part of stroma showing anamorph (*Hirsutella* A-type), with a palisade of phialides, subulate at the base and tapering to a needle-like neck producing narrowly limoniform conidia (bar  = 10 µm); **b**) Ascospores newly released onto agar, distinctly vermiform in shape (bar  = 15 µm); **c**) Ascospore germinating after 24 h on agar, with a needle-like outgrowth (capilliconidiophore) producing a terminal conidium with prominent cap (bar  = 10 µm).

Mycelium densely produced from all orifices and sutures; initially white, silky, becoming ginger to chocolate brown. Stromata single, produced from dorsal pronotum, commonly 5–8 mm, up to 15 mm in length, cylindrical, ginger to dark brown at base, white to pinkish in fertile upper part; fertile region of lateral cushions, 1–3, disc-shaped to hemisphaerical, pale to chestnut-brown, darkening with age, variable in size, averaging 1×0.5 mm, occasionaly up to 2 mm long (Fig. 2a–b). Ascomata immersed to partially erumpent, flask-shaped, 175–260×100–130 µm, with short, exposed neck or ostiole (Fig. 2c–d). Asci 8-spored, hyaline, cylindrical to clavate, (110-) 120–160×(6–) 8–10 µm; cap prominent, 4.0–5.5×3.0–4.5 µm (Fig. 2e). Ascospores multiserriate, hyaline, thin-walled, vermiform, (75–) 80–95 (–115)×2–3 µm, 4–7-septate, sinuous to curved, rarely straight; apex acute, with a rounded base (Fig. 3b).

#### Anamorph

*Hirsutella*-A type [9] only: produced laterally on upper stromata; phialides cylindrical to lageniform, 10×2 µm, tapering to a long neck, 15–25 µm, up to 45 µm; conidia fusiform to narrowly limoniform, averaging 5×1.5 µm, with a distinct tail (Fig. 3a). This anamorph occurred in all species, and, although there may be interspecies variations in morphology, these appear to be slight and were not critically analysed for species separation since other, more critical characters were present. In addition, this stage in the rarely-collected species invariably was in a poor condition.

#### Additional specimens examined

**BRAZIL. Minas Gerais**: Viçosa, Mata do Paraíso, 3 Mar 2010, *H.C. Evans*, *D.P. Hughes & S.L. Elliot*, MAP-53 (paratype IMI 399092)

#### Germination process

The released ascospores germinated within 24 h to produce (1–)3, uniformly straight, extremely narrow (*ca*. 0.2 µm diam) hair-like structures (capilliconidiophores); variable in length, (45–) 60–70 (–80) µm; bearing a single terminal spore (capilliconidium), hyaline, smooth-walled, guttulate, clavate, (7–) 9–11×1.5–2.5 µm, narrowing apically, with age darkening and developing a prominent cap (Fig. 3c).

**Ophiocordyceps camponoti-balzani** H.C. Evans & D.P. Hughes sp. nov.

**IF 550002**

http://www.indexfungorum.org/Names/NamesRecord.asp?RecordID=550002

Type: Brazil. Minas Gerais: Viçosa, Mata do Paraíso, 3 Mar 2010, *S.L. Elliot & D.P. Hughes*, MAP-6, on *Camponotus balzani* Emery (holotype IMI 399090; isotype VIC 31422).

Species *Ophiocordyceps camponoti-rufipedis* similis, sed ascosporae cylindricae, 135–175×4–5 µm, 14–22-septatae, differt (Table 1).

**Table 1:** Comparison of main morphological characters of new *Ophiocordyceps* species.

	Ascospores			Hirsutella Type		
	Shape	Size (µm)	Septation	A	B	C
*O. camponoti-rufipedis*	vermiform	80–95×2–3	4–7	+	-	-
*O. camponoti-balzani*	cylindrical	135–175×4–5	14–22	+	-	+
*O. camponoti-melanotici*	cylindrical	170–210×4–5	27–35	+	-	-
*O. camponoti-novogranadensi*s	filiform	75–95×2.5–3.5	5–10	+	+	-

**Figure 4:**
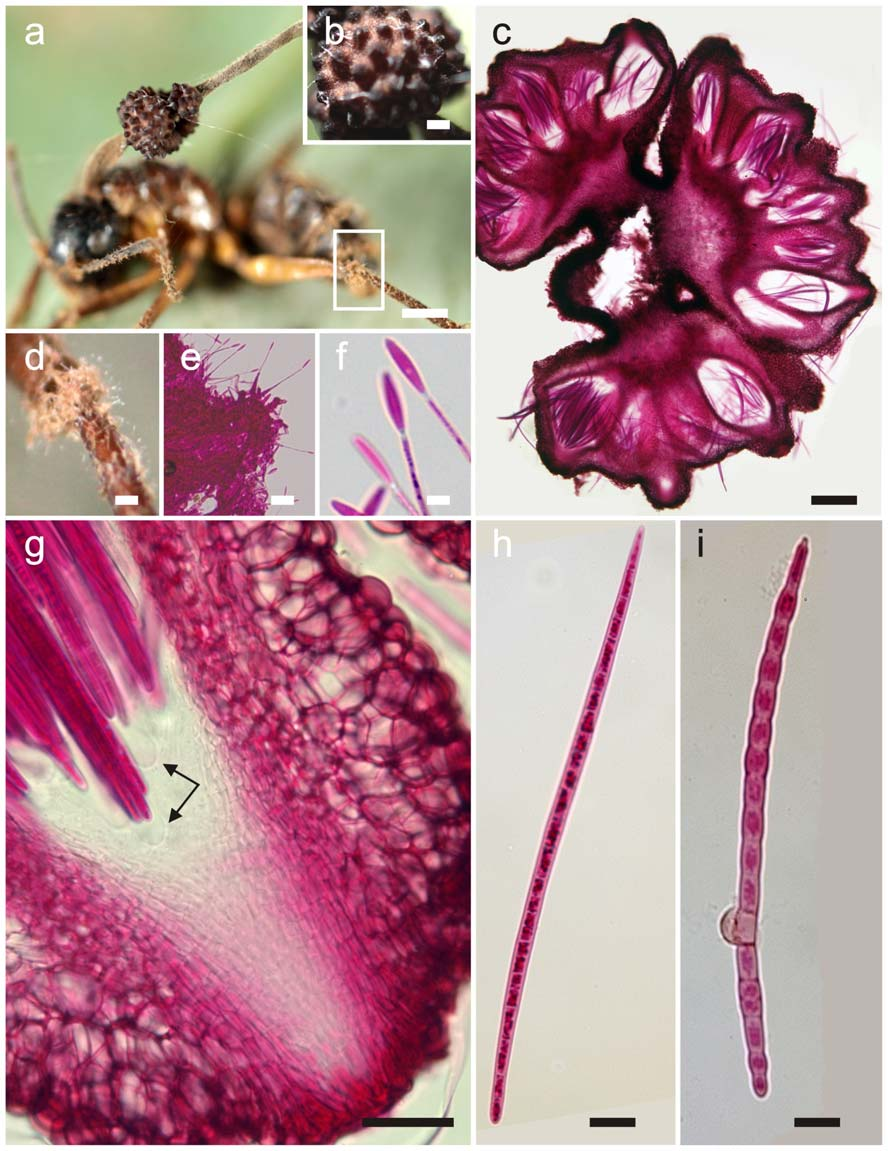
*Ophiocordyceps camponoti-balzani*. **a**) Stroma of *Ophiocordyceps* on *Camponotus balzani* (bar  = 1 mm); **b**) Detail of semi-erumpent ascomata with prominent ostioles (bar  = 0.4 mm); **c**) Section through cushion showing arrangement and semi-erumpent nature of ascomata (bar  = 150 µm); **d**) Close-ups from **a**) of mycelial cushions (sporodochia) on legs and antennae (bar  = 0.2 mm), showing *Hirsutella* C-type phialides (**e**, bar  = 10 µm) and apical conidia (**f**, bar  = 5.0 µm); **g**) Section through ascoma showing prominent ostiole and ascus tips with refractive caps (bar  = 30 µm); **h**) Ascospore, broadly cylindrical, large and multiseptate (bar  = 12.5 µm, compare with **Fig. 3b**); **i**) Ascospore after one month on agar, slightly swollen and producing a lateral swelling, probably a vestigial appressorium (bar  = 12.5 µm).

Mycelium chocolate brown, forming aggregations or cushions around joints, especially on legs and antennae. Stromatal morphology similar to *O. camponoti-rufipedis*, but fertile region dark brown to black when mature, averaging 1.5×1.0 mm (Fig. 4a). Ascomata semi-erumpent, flask-shaped, (350–) 400–450×100–150 (–200) µm, with a pronounced neck (Fig. 4b–c). Asci 8-spored, hyaline, cylindrical, 200–240×12–16 µm; apical cap prominent, (6–) 8–10×6–8 µm (Fig. 4g). Ascospores multiserriate, hyaline, thin-walled, broadly cylindrical, (120–) 135–175×4.0–5.0 µm, 14–22 (–27)-septate, mainly straight or gently curved; rounded at base, tapering to a pointed apex (Fig. 4h).

#### Anamorph

As for *O. camponoti-rufipedis*, *Hirsutella* A-type associated with apical region of stromata. *Hirsutella* C-type ( =  *H. sporodochialis*-type), [8], produced from brown cushions (sporodochia) on leg and antennal joints: phialides subhyaline and subulate at base, robust, 20–25×3–4 µm, tapering to a long, hyaline neck, 30–50×1.0–1.5 µm; conidia hyaline, guttulate, cylindric to fusiform, 12–14×2–3 µm, rounded at apex, truncate at base (Fig. 4d–f).

#### Additional specimens examined

**BRAZIL. Minas Gerais**: Viçosa, Mata do Paraíso, 28 May 2010, *H.C. Evans & D.P.* Hughes, MAP-81 (paratype IMI 399091); Ouro Preto, Parque Estadual de Itacolomi, 4 June 2010, *H.C. Evans & D.P. Hughes*, OP-25 (paratype IMI 399093).

#### Germination process

The majority of ascospores remain unchanged after 28 days on DWA; occasionaly showing cell swellings, especially centrally, with the formation of appressorial-like structures (Fig. 4i).

**Ophiocordyceps camponoti-melanotici** H.C. Evans & D.P. Hughes sp. nov.

**IF 550003**

http://www.indexfungorum.org/Names/NamesRecord.asp?RecordID=550003

Type: Brazil. Minas Gerais: Viçosa, Mata do Paraíso, 27 Apr 2010, *H.C. Evans & D.P. Hughes*, MAP-70, on *Camponotus melanoticus* Emery (holotype IMI 399094; isotype VIC 31425).

Species *Ophiocordyceps camponoti-rufipedis* similis, sed ascosporae cylindricae, 170–210×4–5 µm, 27–35-septatae, differt (Table 1).

**Figure 5:**
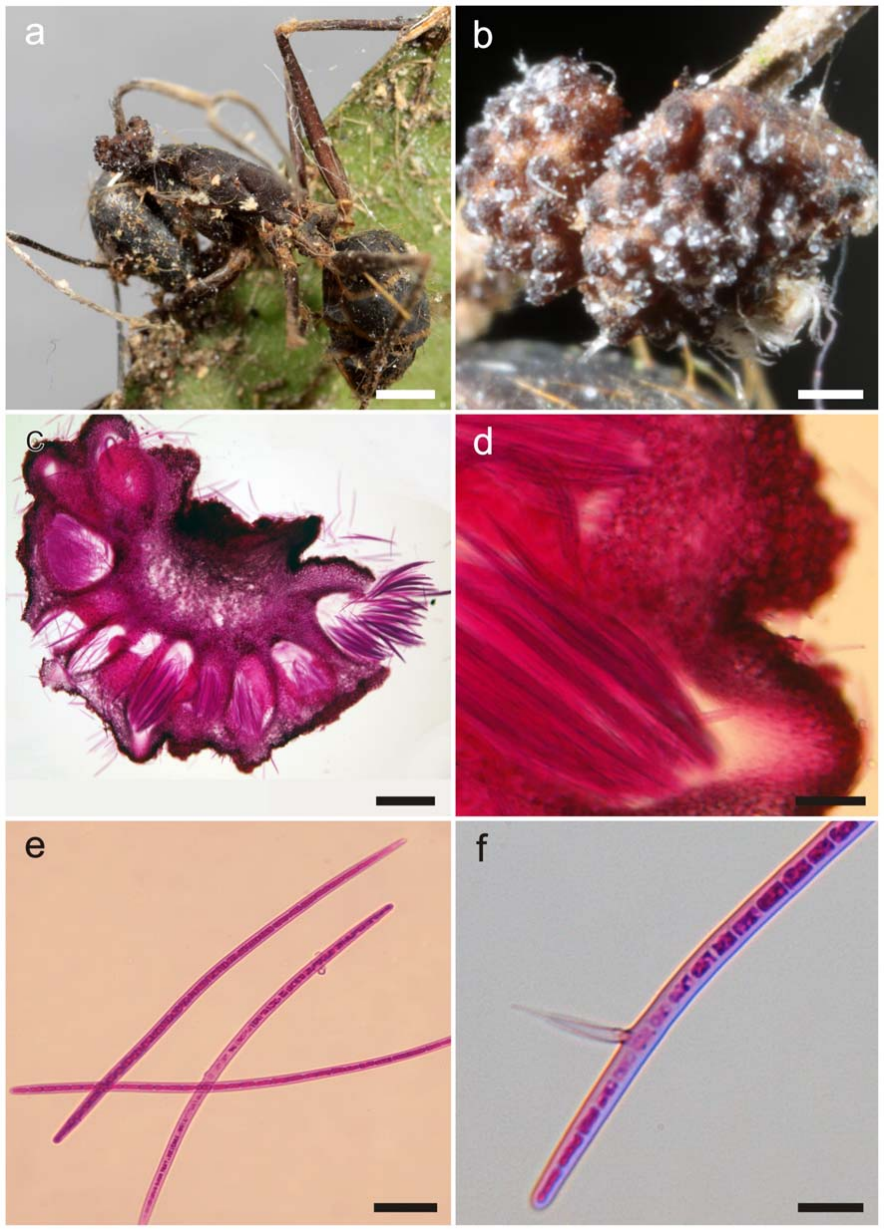
*Ophiocordyceps camponoti-melanotici*. **a**) *Camponotus melanoticus* biting into a leaf, the upper part of the stroma is forked, probably due to damage during growth (bar  = 1 mm); **b**) Detail of ascomatal cushion showing semi-erumpent ascomata with prominent ostioles (bar  = 0.25 mm); **c**) Section through cushion (bar  = 200 µm), with detail of ostiolar region (**d**, bar  = 75 µm); **e**) Ascospores released from ascomata (bar  = 25 µm), and **f**) Ascospore after one month on agar showing solitary phialide (*Hirsutella* A-type, bar  = 10 µm).

Mycelium chocolate to dark brown, relatively sparse. Stromatal morphology similar to *O. camponoti-rufipedis*. Fertile area dark brown to black, 1.3×0.8–1.0 mm (Fig. 5a–b). Ascomata semi-erumpent, flask-shaped, 400–450×100–150 µ µm, with a prominent neck (Fig. 5 c–d). Asci 8-spored, 200–275 (–300)×12–16 µm; apical cap, 8–10×6–8 µm. Ascospores hyaline, thin-walled, broadly cylindrical, 170–210×4–5 µm, 27–35-septate, predominantly straight; truncate at base, apex acute (Fig. 5e).

#### Anamorph

*Hirsutella* A-type only, on upper part of stromata: both specimens (see below) bearing only remnant hymenium on ageing stromata.

Additional specimens: Only two specimens collected; one holotype and one isotype deposited.

#### Germination process

On DWA, most ascospores unchanged after 28 days, minority producing solitary *Hirsutella* phialide (Fig. 5f); on PCA, cells become grossly swollen forming bead-like chains.

**Ophiocordyceps camponoti-novogranadensis** H.C. Evans & D.P. Hughes sp. nov.

**IF 550004**

http://www.indexfungorum.org/Names/NamesRecord.asp?RecordID=550004

Type: Brazil. Minas Gerais: Ouro Preto, Parque Estadual de Itacolomi, 1,000 m, 4 May 2010, *H.C. Evans & D.P. Hughes*, OP-10, on *Camponotus novogranadensis* Mayr (holotype IMI 399095; isotype VIC 31423).

Species *Ophiocordyceps camponoti-rufipedis* similis, sed ascosporae filiformes, 75–95×2.5–3.5 µm, 5–10-septatae, differt (Table 1).

**Figure 6:**
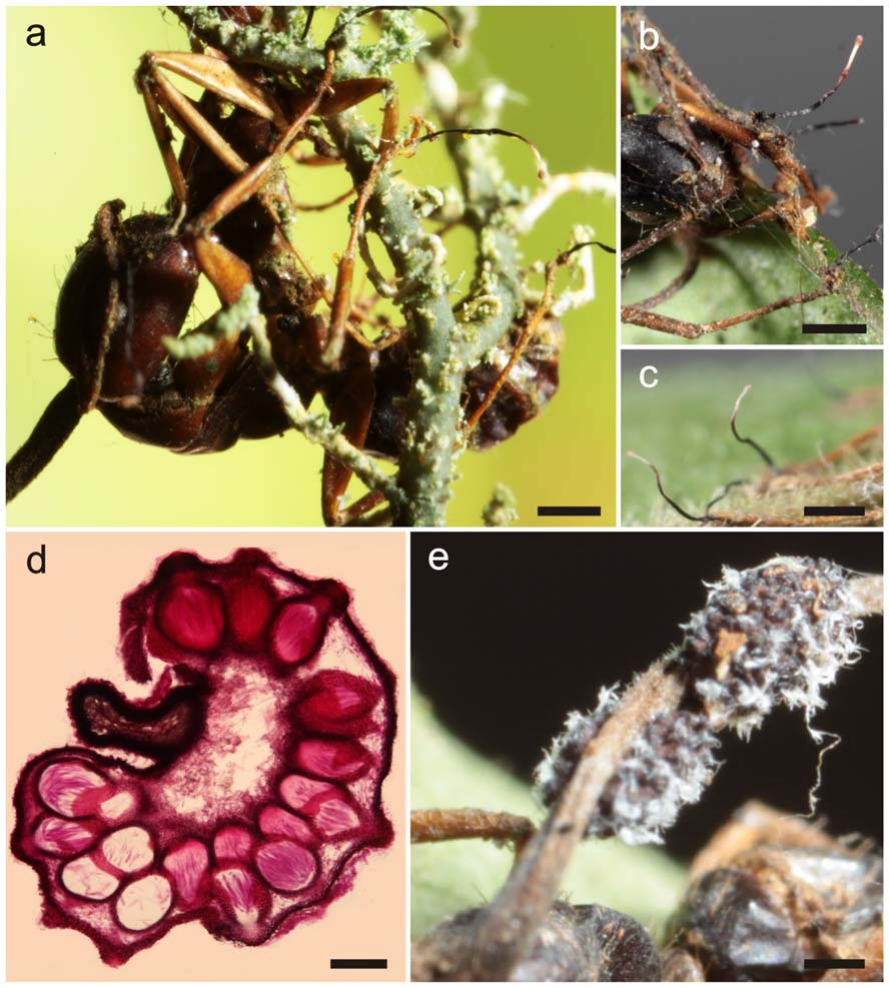
*Ophiocordyceps camponoti-novogranadensis*. **a**) Infected *Camponotus novogranadensis* biting into a lichen epiphyte with anamorph synnemata (*Hirsutella* B-type) arising from feet (bar  = 0.3 mm), and highlighted in **b**) and **c**) (bar  = 0.4 mm); **d**) Section through ascomatal cushion (bar  = 100 µm); **e**) Ascomatal cushions with white masses of discharged ascospores (bar  = 0.3 mm).

**Figure 7:**
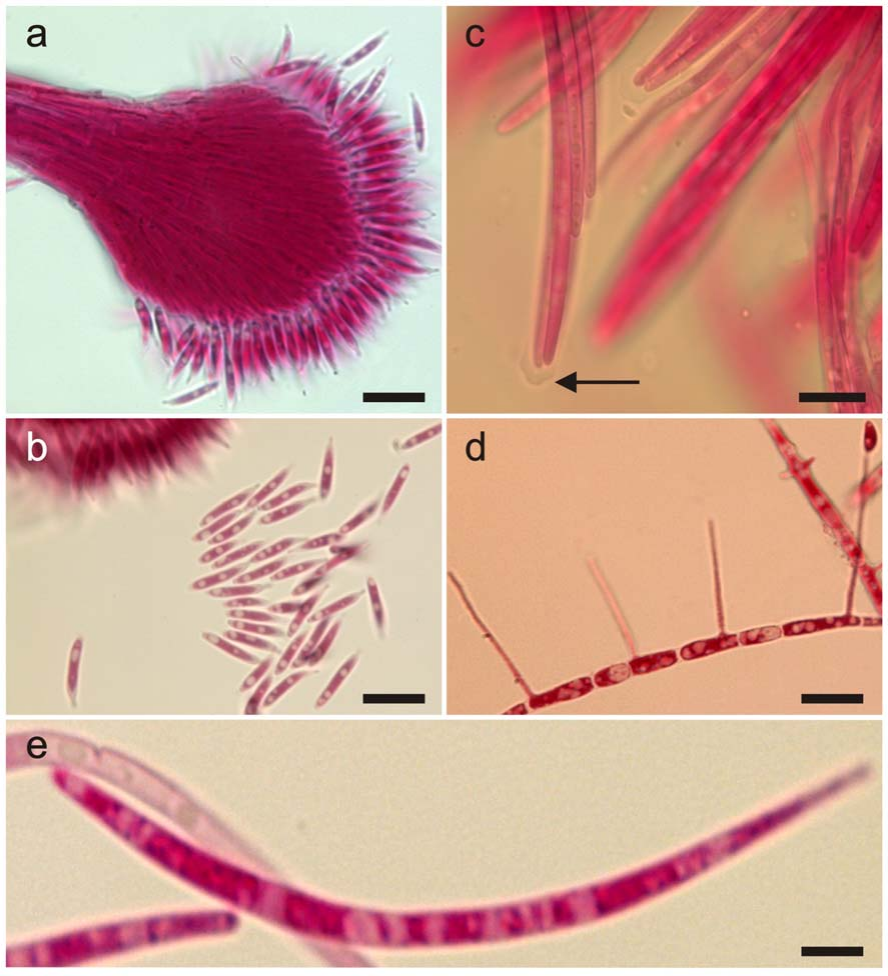
*Ophiocordyceps camponoti-novogranadensis*. **a**) Anamorph (*Hirsutella* B-type) showing detail of conidiogenesis (bar  = 15 µm), and biguttulate conidia (**b**, bar  = 10 µm); **c**) Asci with small but prominent caps (bar  = 10 µm). **d**) Ascospore after 48 h on agar producing four capilliconidiophores (bar  = 10 µm); **e**) Filiform ascospore (bar  = 5 µm).

Mycelium light to chocolate brown, especially dense around feet forming distinctive pads. Stromatal morphology as for *O. camponoti-rufipedis*. Fertile region brown, 0.8–1.0×0.5–0.6 µm. Ascomata semi-erumpent, crowded, 225–250×125–155 µm (Fig. 6d). Asci 8-spored, hyaline, cylindrical, 95–120×9–10 µm, with prominent apical cap, 5–6×3–4 µm (Fig. 7e). Ascospores hyaline, thin-walled, filiform, 75–95×2.5–3.5 µm, 5–10-septate, mainly curved; base rounded, apex acute (Fig. 7c).

#### Anamorph

*Hirsutella* A-type on stromata. *Hirsutella* B-type [9] produced from lower joint/foot on all legs, consisting of solitary, upright synnema (Fig. 6a–c); black, cylindrical, 0.8–1.0 cm, 35–40 µm at base, tapering to 15–18 µm towards apex and broadening into globose head (*ca*. 50 µm diam); phialides terminal, hyaline, subulate, up to 45 µm long, 1.5–2.0 µ µm wide; conidia hyaline, typically biguttulate, narrowly clavate to obclavate, 10–12×(1.0–) 1.5–2.0 µm, truncate at base tapering to drawn out beak-like tip, produced in pinkish, powdery masses (7a–b).

#### Additional specimens examined. BRAZIL

**Minas Gerais**: Ouro Preto, Parque Estadual de Itacolomi, 4 Jun 2010, *H.C. Evans & D.P. Hughes*, OP-24 (paratype IMI 399096).

#### Germination process

Ascospores germinated within 48 h to produce up to 4 capilliconidiophores per spore, 20–25 µm in length, *ca*. 0.2–04 µm wide; capilliconidia hyaline, biguttulate, reniform, (5–) 7–8×2.5–3.0 µm, slightly truncate at base with a mucoid tip or cap (Fig. 7d).

### Summary points

The four new species proposed herein can readily be separated on morphological characteristics (Table 1). In addition, there are marked differences in the germination process with the smaller, more delicate ascospores of *O. camponoti-rufipedis* and *O. camponoti-novogranadensis* producing secondary spores (capilliconidia); whilst the significantly longer and stouter ascospores of *O. camponoti-balzani* and *O. camponoti-melanotici* fail to or only occasionaly germinate *in vitro*, but never produce capilliconidiophores. From these preliminary data, it is tantalising to speculate that each species of the tribe *Camponotini* may be attacked by a distinct species of *Ophiocordyceps*. However, no studies, up until now, have examined living material and, therefore, there are few details of patterns of septation (this is difficult to establish in immature or non-discharged spores) and germination, which are considered here as critical additional characters to separate species. Circumstantial evidence can be gleaned from the literature revealing considerable variation in stromatal and ascospore morphology between collections [1], [2], [8], [11], as well as in anamorph morphology [8]. Indeed, the latter study shows a striking range of synanamorphs associated with species of *Polyrhachis* in Africa. Whether or not this hypothesis is correct will await more in-depth studies using both morphological and molecular data. Some recent collections from North America indicate that this theory may not always hold true but the naming of the new species here, based on the ant host attacked, seems justified and robust enough since there was no evidence of cross infections within overlapping or sympatric ant populations. For this reason, it is considered that the four species described here are distinct from *O. unilateralis* - even though the holotype is effete - because it is associated with a different species of *Camponotus*, purportedly *C. sericeiventris*. Clearly, however, there is an impasse until mature specimens are found on *C. sericeiventris* and a neotype is designated.

### Functional morphology related to host ecology

We put forward the hypothesis that ant ecology drives the functional morphology of the fungal pathogen. Through a combination of behavioural and morphological studies, we may begin to understand the diversity of *O. unilateralis s.l.* at the global level which in turn will allow us to look beyond the ant-infecting *Ophiocordyceps* and ask how specialised are other species of *Ophiocordyceps* attacking members of diverse insect orders from the Orthoptera to Lepidoptera.

## Materials and Methods

### Specimens

Collection of specimens was concentrated in two small areas of remnant, secondary Atlantic rainforest in the south-eastern region of the State of Minas Gerais, Brazil: Mata do Paraíso (MAP), a forest reserve of around 400 ha belonging to the Federal University of Viçosa, *ca*. 700 m a.s.l.; Parque Estadual de Itacolomi, Ouro Preto (OP), *ca*. 150 km west of MAP and 1,000 m a.s.l., comprising a much larger area of mixed forest (>7,500 ha), but with collecting restricted to a fragment of low-canopy, seasonally-flooded forest where high densities of infected ants had been recorded previously [12], (Sérvio Ribeiro, pers. comm., May 2010). This involved a careful inspection of shrubs and tree boles up to *ca*. 2 m and any infected ants plus attached substratum (leaves, small branches and epiphytic lichens) were transferred to sterile containers. Preferably, these were examined the same day for initial identification and the selection of specimens with well developed stromata for morphological studies.

### Morphological studies

For those ant species where abundant material was collected, stalks bearing mature stromata were removed from the host, attached at the base to the lid of a plastic Petri dish – previously smeared with a film of Vaseline – and suspended above a dish containing either distilled water agar (DWA) or potato carrot agar (PCA). Where material was in short supply, the infected ant was attached directly to the lid. These were observed daily to monitor release of ascospores, usually signalled by white spore shadows on the agar surface and/or on the lid. Ejected ascospores were transferred aseptically to fresh DWA plates or to PCA, and incubated (20–25°C) for up to one month to observe the germination process, using an Olympus stereomicroscope.

Subsequently, free-hand sections were made from the stromata using a razor blade. For microscopic examination, material was mounted in 1% acid fuchsin and observed using an Olympus BX51 light microscope fitted with a MicroPublisher 3.3 RTV Q an Olympus E330 imaging camera. A minimum of 50 discharged (mature) ascospores were examined and measured for the comparative morphology (Table 1).

Specimens, together with permanent slides of all the relevant structures, were deposited in Herb IMI (RBG, Kew) with isotype collections held in Herb VIC (Federal University of Viçosa, UFV, Minas Gerais, Brazil). Permits for export and collecting were provided by IBAMA to SLE (23920-1). There were no live animals used in our study.

### Nomenclature

The electronic version of this document in itself does not represent a published work according to the International Code of Botanical Nomenclature, and hence the new names contained in the electronic version are not effectively published under that Code from the electronic edition alone. Therefore, a separate edition of this document was produced by a method that assures numerous identical printed copies, and those copies were simultaneously distributed (on the publication date noted on the first page of this article) for the purpose of providing a public and permanent scientific record, in accordance with Article 29 of the Code. Copies of the print-only edition of this article were distributed on the publication date to herbaria or generally accessible libraries of the following institutions (CABI, Harvard Farlow, CBS, Kew, New York Botanical Garden, USDA Systematic Mycology and Microbiology Laboratory and Mycology Lab, Beijing). The separate print-only edition is available on request from PLoS (Public Library of Science) by sending a request to *PLoS ONE*, Public Library of Science, 1160 Battery Street, Suite 100, San Francisco, CA 94111, USA along with a check for $10 (to cover printing and postage) payable to “Public Library of Science”. In addition, new names contained in this work have been submitted to Index Fungorum (http://www.indexfungorum.org/), from where they will be made available to the Global Names Index.
